# Supplementary material for: Rapid and Convenient Single-Chain Variable Fragment-Employed Electrochemical C-Reactive Protein Detection System
Source: Int J Mol Sci. 2024 Mar 1;25(5):2859. doi: 10.3390/ijms25052859 (PMC10931773; doi:10.3390/ijms25052859)
Supplement: Supplementary file 1 [file ijms-25-02859-s001.zip › ijms-2811317-supplementary.pdf]

Supplementary information

Rapid and Convenient Single-Chain Variable Fragment-Employed Electrochemical C-Reactive Protein Detection System

Daimei Miura [1,†], Saki Motohashi [1,†], Ayaka Goto [1], Hayato Kimura [1], Wakako Tsugawa [1], Koji Sode [2], Kazunori Ikebukuro [1] and Ryutaro Asano [1,3,*]

[1] Department of Biotechnology and Life Science, Graduate School of Engineering, Tokyo University of
Agriculture and Technology, 2-24-16 Naka-cho, Koganei 184-8588, Japan; daimei@go.tuat.ac.jp (D.M.);
motohashisaki@gmail.com (S.M.); gotoaya@st.go.tuat.ac.jp (A.G.); mich8810eal@gmail.com (H.K.);
tsugawa@cc.tuat.ac.jp (W.T.); ikebu@cc.tuat.ac.jp (K.I.)

[2] Joint Department of Biomedical Engineering, University of North Carolina at Chapel Hill and North
Carolina State University, Chapel Hill, NC 27599, USA; ksode@email.unc.edu

[3] Institute of Global Innovation Research, Tokyo University of Agriculture and Technology,
3-8-1 Harumi-cho, Fuchu 183-8509, Japan

* Correspondence: ryutaroa@cc.tuat.ac.jp

† These authors contributed equally to this work.

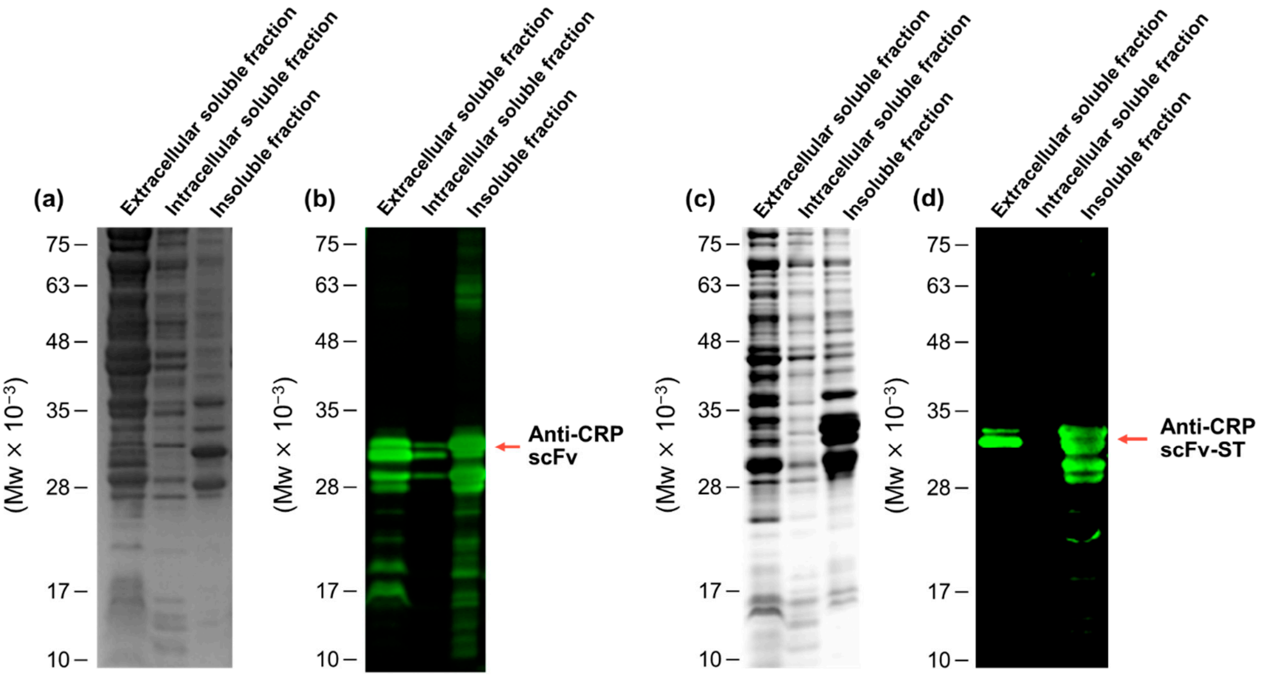

Figure S1 SDS-PAGE and Western blotting analysis for confirmation of expression of (a, b) anti-CRP scFv and (c, d) anti-CRP scFv-ST).

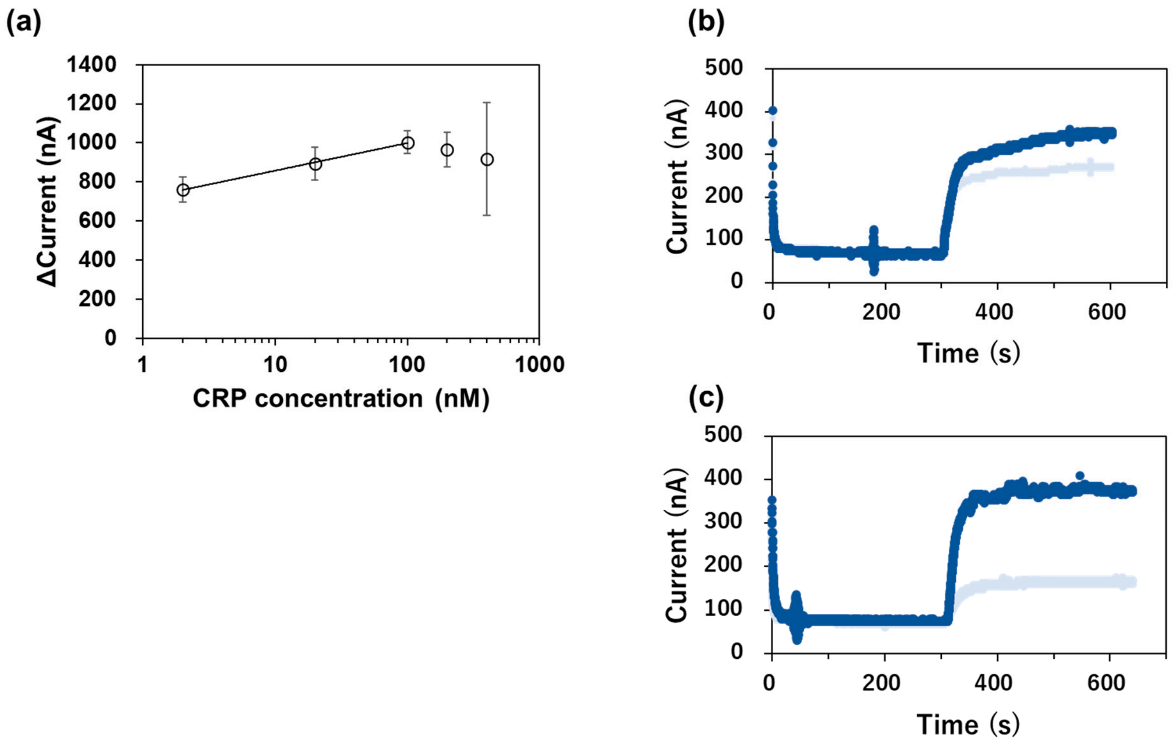

Figure S2 (a) A calibration curve of the electrochemical detection of CRP detection using our previous protocol (n = 3). Electrochemical CRP detection (b) without and (c) with washing after incubation with bivalent AEC, CRP, or scFv-immobilized magnetic beads (n = 1).

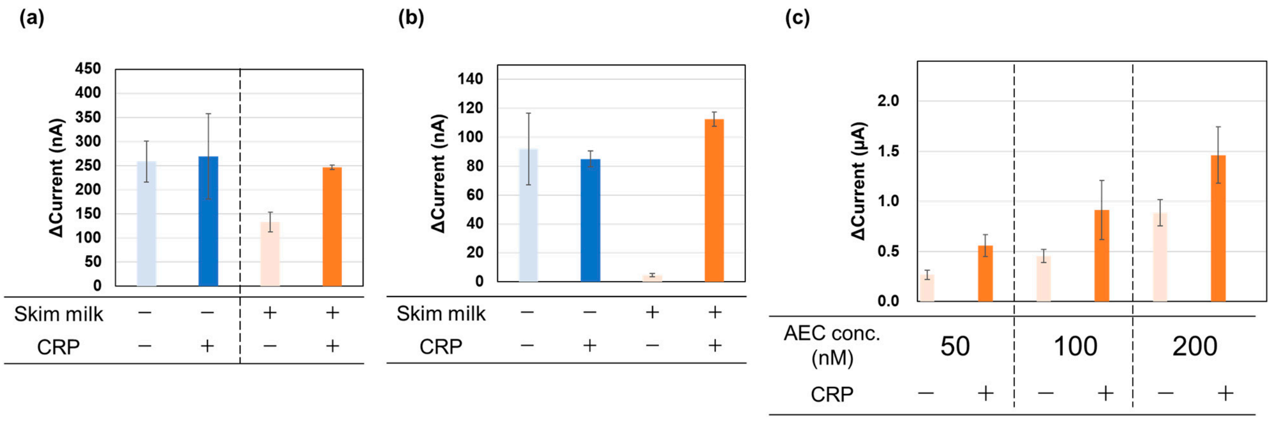

Figure S3 Investigation of the conditions for electrochemical detection of CRP. (a) The effect of skim milk during the incubation of AEC and CRP with scFv-captured magnetic beads on the prevention of non-specific binding of the bivalent AEC. (b) The effect of a washing procedure and addition of skim milk during the incubation of AEC and CRP with scFv-captured magnetic beads. (c) The effect of the concentration of the bivalent AEC. All data are presented as mean ± S.D. (n = 3).

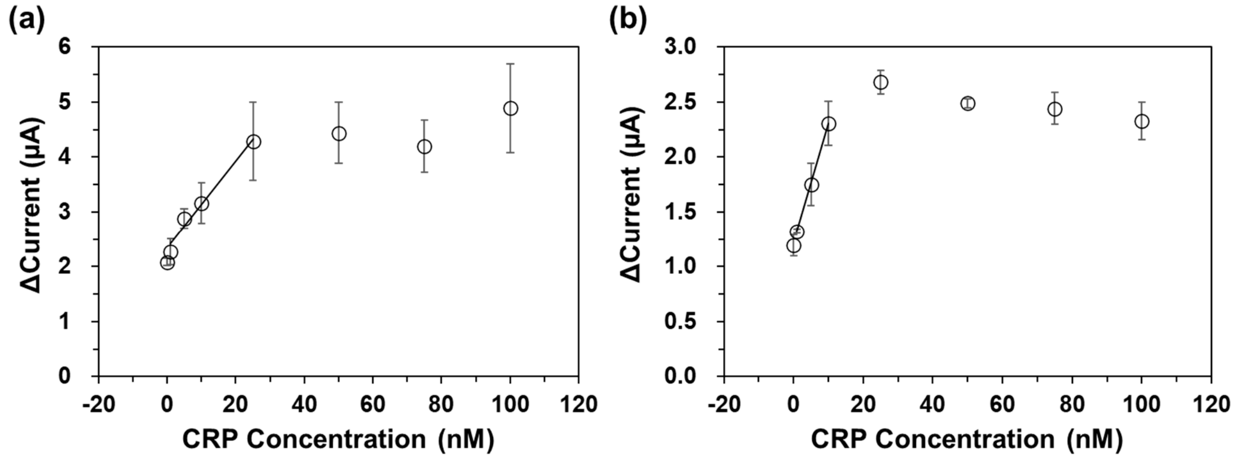

Figure S4 Calibration curves of detection (a) in buffer and (b) in serum. All data are presented as mean ± S.D. (n = 3).

Table S1 Amino acid sequences of the proteins used in this study.

Protein	Sequence
**Anti-CRP scFv**	MKYLLPTAAAGLLLLAAQPAMAHMASQVQLQQSGAELVKPGASVKLSCTASGFNIKDYYMHWVKQRTEQGLEWIGRIDPEDGETKYAPKFQGKATITADTSS NTAYLQLSSLTSEDTAVYYCARGYYGSEAMDYWGQGTSLTVSSGGGGSGGGGSGGGGSTGSIVMTQSHKFMSTSVGDRVSITCKASQDVNTAVAWYQQK PGQSPKLLIYWASTRHTGVPDRFTGSGFGTDYTLTISSVQAEDLALYYCQQHYSTPWTFGGGTKLEIKRADAAPKLAAAAEQKLISEEDLNLGGGMRGSHHHH HH
**Anti-CRP scFv-ST**	MKYLLPTAAAGLLLLAAQPAMAHMASQVQLQQSGAELVKPGASVKLSCTASGFNIKDYYMHWVKQRTEQGLEWIGRIDPEDGETKYAPKFQGKATITADTSS NTAYLQLSSLTSEDTAVYYCARGYYGSEAMDYWGQGTSLTVSSGGGGSGGGGSGGGGSTGSIVMTQSHKFMSTSVGDRVSITCKASQDVNTAVAWYQQK PGQSPKLLIYWASTRHTGVPDRFTGSGFGTDYTLTISSVQAEDLALYYCQQHYSTPWTFGGGTKLEIKRADAAPKLAAAGSGAHIVMVDAYKPTKAEQKLISEE DLNLGGGMRGSHHHHHH
**SC**	MGAMVDTLSGLSSEQGQSGDMTIEEDSATHIKFSKRDEDGKELAGATMELRDSSGKTISTWISDGQVKDFYLYPGKYTFVETAAPDGYEVATAITFTVNEQGQ VTVNGKATKGDAHIHHHHHH
**GDH-SC**	MNTTTYDYIVVGGGTSGLVVANRLSENPDVSVLLLEAGASVFNNPDVTNANGYGLAFGSAIDWQYQSINQSYAGGKQQVLRAGKALGGTSTINGMAYTRAED VQIDVWQKLGNEGWTWKDLLPYYLKSENLTAPTSSQVAAGAAYNPACNGKEGPLKVGWSGSLASGNLSVALNRTFQAAGVPWVEDVNCGKMRGFNIYPST LDVDLNVREDAARAYYFPYDDRKNLHLLENTTANRLFWKNGSAEEAIADGVEITSADGKVTRVHAKKEVIISAGALRSPLILELSGVGNPTILKKNNITPRVDLPT VGENLQDQFNNGMAGEGYGVLAGASTVTYPSISDVFGNETDSIVASLRSQLSDYAAATVKVSNGHMKQEDLERLYQLQFDLIVKDKVPIAEILFHPGGGNAVS SEFWGLLPFARGNIHISSNDPTAPAAINPNYFMFEWDGKSQAGIAKYIRKILRSAPLNKLIAKETKPGLSEIPATAADEKWVEWLKANYRSNFHPVGTAAMMPR SIGGVVDNRLRVYGTSNVRVVDASVLPFQVCGHLVSTLYAVAERASDLIKEDAKSAGSGGAMVDTLSGLSSEQGQSGDMTIEEDSATHIKFSKRDEDGKELAG ATMELRDSSGKTISTWISDGQVKDFYLYPGKYTFVETAAPDGYEVATAITFTVNEQGQVTVNGKATKGDAHILEHHHHHH
**SC-GDH-SC**	MGSSHHHHHHSSGLVPRGSGAMVDTLSGLSSEQGQSGDMTIEEDSATHIKFSKRDEDGKELAGATMELRDSSGKTISTWISDGQVKDFYLYPGKYTFVETAA PDGYEVATAITFTVNEQGQVTVNGKATKGDAHIGSGHMNTTTYDYIVVGGGTSGLVVANRLSENPDVSVLLLEAGASVFNNPDVTNANGYGLAFGSAIDWQY QSINQSYAGGKQQVLRAGKALGGTSTINGMAYTRAEDVQIDVWQKLGNEGWTWKDLLPYYLKSENLTAPTSSQVAAGAAYNPACNGKEGPLKVGWSGSLA SGNLSVALNRTFQAAGVPWVEDVNCGKMRGFNIYPSTLDVDLNVREDAARAYYFPYDDRKNLHLLENTTANRLFWKNGSAEEAIADGVEITSADGKVTRVHA KKEVIISAGALRSPLILELSGVGNPTILKKNNITPRVDLPTVGENLQDQFNNGMAGEGYGVLAGASTVTYPSISDVFGNETDSIVASLRSQLSDYAAATVKVSNG HMKQEDLERLYQLQFDLIVKDKVPIAEILFHPGGGNAVSSEFWGLLPFARGNIHISSNDPTAPAAINPNYFMFEWDGKSQAGIAKYIRKILRSAPLNKLIAKETKP GLSEIPATAADEKWVEWLKANYRSNFHPVGTAAMMPRSIGGVVDNRLRVYGTSNVRVVDASVLPFQVCGHLVSTLYAVAERASDLIKEDAKSAGSGGAMVD TLSGLSSEQGQSGDMTIEEDSATHIKFSKRDEDGKELAGATMELRDSSGKTISTWISDGQVKDFYLYPGKYTFVETAAPDGYEVATAITFTVNEQGQVTVNGK ATKGDAHILEHHHHHH
